# Epigenetic Regulation in Primary CNS Tumors: An Opportunity to Bridge Old and New WHO Classifications

**DOI:** 10.3390/cancers15092511

**Published:** 2023-04-27

**Authors:** Danielle D. Dang, Jared S. Rosenblum, Ashish H. Shah, Zhengping Zhuang, Tara T. Doucet-O’Hare

**Affiliations:** 1 Neuro-Oncology Branch, Center for Cancer Research, National Cancer Institute, National Institutes of Health, Bethesda, MD 20892, USA; 2 Section of Virology and Immunotherapy, Department of Neurosurgery, Leonard M. Miller School of Medicine, University of Miami, Miami, FL 33136, USA

**Keywords:** SWI/SNF, chromatin remodeling, central nervous system tumors, endogenous retroviruses, HERVK, atypical teratoid rhabdoid tumor, glioblastoma, meningioma, schwannoma, World Health Organization

## Abstract

**Simple Summary:**

In this review, the authors re-evaluate the fifth edition of the World Health Organization Classification of Central Nervous System tumors in light of recent advances in epigenetic tools and evidence of the critical nature of endogenous retroviruses in tumorigenesis. The data systematically presented herein demonstrates that tumors with histopathologic heterogeneity and dichotomous clinical behaviors more often harbor chromatin remodeling defects and/or associated aberrant endogenous retrovirus expression. The authors believe these observations warrant further investigation as they could potentially lead to a deeper understanding of tumor biology and more translationally relevant tumor stratification.

**Abstract:**

Originally approved in 1979, a specific grading classification for central nervous system (CNS) tumors was devised by the World Health Organization (WHO) in an effort to guide cancer treatment and better understand prognosis. These “blue books” have since undergone several iterations based on tumor location, advancements in histopathology, and most recently, diagnostic molecular pathology in its fifth edition. As new research methods have evolved to elucidate complex molecular mechanisms of tumorigenesis, a need to update and integrate these findings into the WHO grading scheme has become apparent. Epigenetic tools represent an area of burgeoning interest that encompasses all non-Mendelian inherited genetic features affecting gene expression, including but not limited to chromatin remodeling complexes, DNA methylation, and histone regulating enzymes. The SWItch/Sucrose non-fermenting (SWI/SNF) chromatin remodeling complex is the largest mammalian family of chromatin remodeling proteins and is estimated to be altered in 20–25% of all human malignancies; however, the ways in which it contributes to tumorigenesis are not fully understood. We recently discovered that CNS tumors with SWI/SNF mutations have revealed an oncogenic role for endogenous retroviruses (ERVs), remnants of exogenous retroviruses that integrated into the germline and are inherited like Mendelian genes, several of which retain open reading frames for proteins whose expression putatively contributes to tumor formation. Herein, we analyzed the latest WHO classification scheme for all CNS tumors with documented SWI/SNF mutations and/or aberrant ERV expression, and we summarize this information to highlight potential research opportunities that could be integrated into the grading scheme to better delineate diagnostic criteria and therapeutic targets.

## 1. Introduction

The World Health Organization (WHO) periodically releases specialty-specific updates to a systematic classification of pathologies to guide clinical practice. These periodic updates are released with the intent of bridging the gap between new basic scientific and clinical discoveries and patient care. The last update for neuro-oncology was released in 2021, bringing us to the fifth edition. These WHO classification schemes have a long and rich history, reflecting the evolution of our understanding of the biological nature of tumors and the development of new diagnostic and therapeutic tools.

The evolution of the WHO classification over time has paralleled the concomitant advances in diagnostic technology. In 1952, the first WHO classification guide for tumors of all organ systems was released, and tumor stratification was based on anatomic site, histopathologic morphology, and a resulting “grade” of malignancy from which to infer prognosis [1]. Although subsequent schemas have been released and updated based on advances in our understanding of tumor biology, this initial framework has continued to be the enduring foundation of the WHO classification. In the 1970s, the WHO classification was updated to include further histopathologic distinctions reflecting a deeper understanding of tumor biology gained through immunohistochemistry and electron microscopy [1]. For the next several decades, these techniques remained the cornerstone for tumor classification, with second and third editions released in 1979 and 2000, respectively. The first edition of the classification scheme specific for central nervous system (CNS) tumors, henceforth known as the “blue books”, however, was published in 1979 by an international committee of expert neuropathologists and neuro-oncologists after a decade of contentious review [1]. With the advent of molecular biology and the ongoing attempts to formulate prognostically meaningful grades, a fourth edition was released in 2016. This edition elaborated on the first examples of standardized molecular profiling in CNS tumors, including the variant status of isocitrate dehydrogenase 1 and 2 (IDH1/2) and the methylation status of 0(6)-methylguanine-DNA methyltransferase (MGMT) [2,3].

The most recent edition (WHO CNS 5) of the schema, which was released in 2021, saw the largest translational shift of focus in tumor classification in several decades. While this recent classification still hinges on the foundational morphologic framework, additional critical criteria for diagnosing and categorizing tumors have been updated to include epigenetic mechanisms that reflect cellular diversity and heterogenous behaviors previously unrecognized in many of these tumor types [4,5].

The paradigm shift in the most recent WHO classification focused on epigenetic mechanisms, which may be grouped into four categories [5]. Those four categories include DNA methylation, histone modification, non-coding RNA molecules, and chromatin remodeling. In reviewing the most recent schema, we found that there appeared to be a tendency of tumors with chromatin remodeling defects, especially the SWItch/sucrose non-fermentable (SWI/SNF) complex, to have increased cellular diversity, a predilection for younger populations or a bi-modal age distribution, and dichotomous clinical behavior in CNS tumors [5]. SWI/SNF deficiency leads to significant changes in the epigenetic control of the genome and can allow for the continual expression of developmental genes post-development or lead to the reactivation and expression of developmental genes in a differentiated cell [6,7]. Epigenetic modifications of DNA in cancer, or heritable changes in gene expression that are not attributable to alterations in DNA sequence, have emerged as a new potential source of novel biomarkers for early cancer detection, prognosis, and presented an opportunity for the development of targeted treatments [8]. Loss of function (LOF) of the SWI/SNF complex can provide opportunities for the development of additional treatments that take advantage of the synthetic lethality that occurs when the perturbation of two genes simultaneously results in loss of viability; however, the disruption of only one of the genes results in survival [9]. Further, we have recently shown that LOF of SWI/SNF proteins can lead to the activation of endogenous retroviruses (ERVs) such as human endogenous retrovirus K (HERV-K, sub-type HML-2) [10]. Normally, ERV expression is tightly spatially and temporally controlled during development, and its expression is significantly downregulated as cells differentiate and mature; therefore, ERV expression provides an excellent target for tumor treatment [10,11,12,13,14,15,16,17]. Potential mechanisms that allow for aberrant expression of ERVs, such as LOF of the SWI/SNF complex, provide a unique opportunity for a combinatorial approach to the treatment of tumors with few targeted treatment options. For example, tumors such as atypical teratoid rhabdoid tumors (AT/RT) and glioblastoma (GBM) have a heterogeneous histopathologic composition that may not be easily encompassed under one categorical term, as is reflected in their clinical behavior and prognosis. To date, we have grouped tumors together that may not belong together solely based on appearance, anatomic location, or presumed cell of origin; however, the clinical behavior of these tumors suggests nuanced distinctions that we can now appreciate with epigenetic profiling.

In our previous work, we evaluated AT/RT, which is a tumor with a loss of function (LOF) of SWI/SNF-related matrix-associated, actin-dependent regulator of chromatin subfamily B member 1 (*SMARCB1*), a core subunit of the SWI/SNF complex, that has a bi-modal age distribution and an aggressive histopathologic appearance but a seemingly unpredictable prognosis [18]. While overall AT/RT has a dismal prognosis, there have been limited reports of groups of long-term survivors with this tumor, suggesting that a subset of these tumors may behave differently than the typical course [18]. In our study of AT/RT patient-derived tumor cell lines and patient tumor tissue, we found that the expression of human endogenous retrovirus K (HERV-K, sub-type HML-2) was facilitated by the LOF of *SMARCB1* and that the expression of HML-2 proteins was critical for tumorigenesis and the maintenance of pluripotency [10]. Expression of ERV proteins has been detected in many tumor types, where possible pathogenic mechanisms include activation of the long terminal repeat element into an oncogene with downstream targets such as MYC and vesicular release of viral particles to neighboring cells to maintain pluripotency [10,11,15]. The precise role of ERV proteins in CNS tumorigenesis, however, represents a critical area of active investigation, with additional exploration required for validation. In light of these preliminary mechanistic findings, ERVs have also recently been explored as a potential therapeutic target for difficult-to-treat tumors [10,19,20]. Our finding of HML-2 activation in AT/RT tumors subsequently prompted us to investigate other primary CNS tumors to evaluate the relationship between defects in chromatin remodeling and endogenous retroviruses for their putative role in tumorigenesis and their potential to further stratify existing classification schemas and better predict tumor behavior.

## 2. Materials and Methods

The 5th edition of the WHO Classification of CNS Tumors (WHO CNS 5) was utilized to systematically identify all primary non-syndromic CNS tumors and to build a database mirroring the classification scheme by tumor categories and subcategories [4,5]. Two authors independently screened each tumor entity for the inclusion of epigenetic mutations documented in the classification. All genes implicated in the tumorigenesis of each tumor were analyzed via the National Library of Medicine for relevant epigenetic function and/or interaction. A subsequent database search in PubMed for human and/or non-human endogenous retrovirus expression in each entity followed. A third author resolved potential discrepancies. Data abstraction additionally included the WHO grade, specific type of known SWI/SNF defect, alternative genetic and/or epigenetic alterations involved in tumorigenesis, and subtype of endogenous retrovirus expressed if previously studied. This process was iteratively completed for each tumor type and is summarized in Supplementary Table S1. Data were excluded if no primary literature, including mechanistic proof of epigenetic alteration or ERV expression, could be retrieved or if implicated genes were only indirectly associated with epigenetic pathways of interest, thereby removing the potential for inferral bias. The authors qualitatively reviewed the data for histopathologic and clinical trends among various tumor groups, including those with SWI/SNF defects, ERV expression, both SWI/SNF defects and ERV expression, genetic and/or epigenetic variants with direct SWI/SNF interaction, and tumors without either characteristic.

## 3. A Systematic Review of Primary CNS Tumors with SWI/SNF Defects and/or Endogenous Retroviral Activation

The WHO CNS 5 is comprised of 100 distinct tumor types based on 11 categories stratified by differentiated cell of origin or anatomic region of the CNS. Upon expansion of the meningioma and germ cell tumor categories into their recognized, distinct histopathologic subtypes, a total of 121 tumors resulted. Of all the tumors in the classification schema, 24 contain known alterations in SWI/SNF subunits. These alterations occurred across 10 discrete subunits of the SWI/SNF chromatin remodeling complex (Table 1). Alpha-thalassemia/mental retardation, X-linked (ATRX) protein represented the most commonly involved variant subunit, followed by SMARCB1, SWI/SNF-related matrix-associated, actin-dependent regulator of chromatin subfamily A member 4 (SMARCA4), AT-rich interaction domain 1A (ARID1A), SWI/SNF-related matrix-associated, actin-dependent regulator of chromatin subfamily A member 2 (SMARCA2), polybromo-1 (PBRM1), SWI/SNF-related matrix-associated, actin-dependent regulator of chromatin subfamily E member 1 (SMARCE1), BRD4 interacting chromatin remodeling complex associated protein (BICRA), AT-rich interaction domain 2 (ARID2), and AT-rich interaction domain 1B (ARID1B). These tumors appeared across multiple categories of terminal differentiation of cell lineage as well as those with variable subsets of clinical behaviors, including gliomas/glioneuronal/neuronal, embryonal, pineal, cranial/paraspinal, meningioma, mesenchymal/non-meningothelial, and germ cell tumors. Further, within these categories, SWI/SNF LOF mutations seemed to correlate predominantly with tumors exhibiting bimodal age distribution, malignant phenotypes, anatomically midline locations, and tumorigenesis that occurs in development regardless of WHO grade. When these mutations were noted in tumors considered “low grade”, due to a low risk of metastatic spread or tumor recurrence, notable histopathologic features that correlate with more aggressive phenotypes were evident [4,5]. For example, in pilocytic astrocytomas and desmoplastic infantile ganglioglioma/desmoplastic infantile astrocytoma, both WHO grade 1 lesions, SMARCB1 and ATRX mutations were documented features only when those tumors featured anaplastic cells. In chordoma, a focally aggressive developmental lesion without a reported WHO grade yet considered “low grade” due to rare metastatic spread, homozygous deletion of SMARCB1 was a hallmark of the poorly differentiated subtype, a rare variant with a worse prognosis in comparison to the conventional chondroid histopathologic subtype. Of the 17 tumors with a reported WHO grade, 12 tumors denoted a high-grade status of 3 or 4, indicating malignant tumor behavior, high risk of recurrence, and poor clinical outcome. The remaining tumors without a WHO grade were notably too rare to render such a distinction (i.e., CNS tumor with BCOR internal tandem repeats) or were considered malignant by conventionally aggressive histopathology, clinical tumor behavior, and prognosis (i.e., malignant peripheral nerve sheath tumor (MPNST)) [4,5].

In our exploration of the literature, we found that human ERVs were implicated in the tumorigenesis of 26 primary CNS tumors, including all meningioma histopathologic subtypes when concomitantly negative for a Merlin mutation (Table 2). Within the human ERVs, HERV-K was most commonly expressed. Non-human ERV expression has been found in a number of studies with primary CNS tumors in animals, of which avian leukosis virus (ALV) was the most common, with additional reporting of tumors caused by repetitive elements endogenized in chickens, mice, cats, and sheep. The importance of ERVs in these primary CNS tumors has been well established in animal models in which retroviruses capable of endogenization have been directly injected into the CNS and resulted in tumor development [72,73]. In parallel, non-human ERVs have naturally contributed to tumorigenesis in wild or domesticated animals outside of the aforementioned research studies [66,74,75,76,77]. Interestingly, we found that there appeared to be some overlap between defects in chromatin remodeling and ERV expression and dichotomous clinical behavior, such as bimodal age distribution and opposing prognosis. While tumors with ERV expression were not restricted by cell of origin or anatomic region, appearing in nine of the 11 tumor categories, the pattern of expression trended with age distribution and malignant histopathologic features. Tumors with ERV expression tended to either affect younger populations or present with a bimodal age distribution. These trends were further strengthened when investigating the subset of tumors that demonstrate both LOF SWI/SNF mutations and ERV expression as contributors to tumorigenesis, of which a total of six tumor types are known (Table 3).

Fifty-three primary CNS tumors had documentation of twenty-eight distinct alternative genetic and/or epigenetic pathways that demonstrate direct protein-protein interaction with the mammalian SWI/SNF chromatin remodeling complex (Table 4). Thirteen entities also shared known primary SWI/SNF subunit variants. Commonly represented epigenetic interactions included histone point mutations affecting chromatin remodeling function via polycomb repressive complex (PRC), direct mutation of PRC2 subunit proteins (EZHIP, SUZ12, EED), transcriptional activators and/or repressors with intrinsic histone acetyltransferase activity (YAP, EP300), and regulators of small non-coding RNA molecules (LIN28A, DICER1). Genetic mutations with direct interaction with SWI/SNF subunits primarily emphasized either transcription factors critical to cell cycle regulation, cell differentiation, and development (*TP53, MYC, RB1*) and/or genes involved in developmental signal transduction (*WNT, SHH, NOTCH*). This cohort of tumors was also associated with many malignant phenotypes predominantly affecting younger populations, particularly within the glioma/glioneuronal/neuronal, embryonal, pineal, meningioma, and mesenchymal categories. Further, a total of 13 of these tumors harbored documented aberrant ERV expression, of which 10 tumors did not share a known SWI/SNF mutation, including the majority of germ cell tumor subtypes, atypical and malignant meningioma subtypes, diffuse large B-cell lymphoma (DLBCL), primary CNS melanoma, and Ewing’s sarcoma (Supplementary Table S2).

Finally, forty-six primary CNS tumors across eight distinct tumor categories exhibited neither LOF SWI/SNF function, alternative genetic/epigenetic mutations with direct SWI/SNF interaction, nor aberrant ERV expression per investigation to date (Table 5). This group was dominated by the entire category of glioneuronal and neuronal tumors, which generally harbor well-established genetic mechanisms and incur a predictable clinical course, including treatment strategy and favorable prognosis. Of the 38 entities that are denoted with a WHO grade, 24 tumors had a low grade (1 or 2) rating, signifying a benign clinicopathologic course and a low risk of tumor recurrence. Only two tumor types, including embryonal tumors with either a very specific genetic mutation (FOXR2) or no known genetic mechanism to date, were classified as WHO grade 3–4 in this group. This cohort also featured several tumor types derived from more mature cell differentiation, including but not limited to pineocytoma, pituicytoma, various lymphomas, and low-grade gliomas, glioneuronal, and neuronal tumors, a feature rarely encountered in tumors that harbored both SWI/SNF LOF mutations and aberrant ERV expression.

## 4. Illustrative CNS Tumors with Tumorigenesis Characterized by Both SWI/SNF Mutations and Endogenous Retroviral Expression

### 4.1. Gliomas, Glioneuronal Tumors, and Neuronal Tumors

Neuronal tumors comprise 1% of all primary brain tumors, generally have favorable clinical outcomes, and can be addressed effectively with surgical removal [143]. These tumors are described histopathologically by the inclusion of mature neuronal cell tumors (e.g., gangliocytoma, dysplastic cerebellar gangliocytoma, and central neurocytoma) and mixed neuronal-glial tumors (e.g., ganglioglioma, desmoplastic infantile ganglioglioma, dysembryoplastic neuroepithelial tumor, and ganglioneuroma) [143]. The clinical behavior of gliomas is often more dependent on their genetics as compared to their histology; however, in glioneuronal and neuronal tumors, histology is central for diagnosis and treatment since these tumors follow a more clinically predictable prognosis [5,144,145]. One of the main ways WHO CNS 5 has restructured the classification of diffuse gliomas is by separating them into adult-type versus pediatric-type and low-grade versus high-grade, with the latter based predominantly on molecular genetic profiling such as IDH1/2 mutation status [4,5,145]. Further, by these new criteria, glioblastoma is universally IDH WT, with a clear distinction from WHO grade 4 IDH mutant astrocytomas [5]. However, even with these additional distinctions between tumor types, there remain subsets of tumors within individual diagnoses that do not adhere to the typical behavior associated with the assigned WHO grade. Within this large, heterogeneous category of CNS primary tumors, epigenetic profiling represents an additional means of investigating these discrepancies and reconciling them. For example, alterations in the SWI/SNF pathway have been found in gliomagenesis as well as aberrant ERV expression [14,15,17,24,25,33,146,147]. This targeted treatment based upon individual tumor profiling may provide a unique therapeutic opportunity for tumors that have historically been treated by non-specific means, which harbor their own potential for inducing tumorigenesis, such as chemotherapy and radiation [148,149].

ATRX (alpha thalassemia/mental retardation syndrome X-linked) is a chromatin remodeling protein contained within the SWI/SNF superfamily of chromatin remodeling proteins and acts as a histone chaperone protein that loads histones onto telomeres to maintain heterochromatin [150,151,152,153,154]. ATRX interacts and forms a complex with Death Domain-Associated Protein (DAXX) to deposit H3.3, a histone variant, at nucleosomes [152]. In a 2012 study of pediatric GBM, somatic mutations in the H3.3-ATRX-DAXX chromatin remodeling pathway were found in 44% (21/44) of tumors; however, under the new WHO CNS 5 criteria, two of the tumors included in this analysis would not be classified as glioblastoma due to IDH mutations [5,33]. Despite this distinction in classification, regardless of IDH mutation status, all these tumors had defects in the H3.3-ATRX-DAXX chromatin remodeling pathway. Considering this, this suggests that there may be differences in the chromatin landscape that we do not yet appreciate that may lead to alterations in ERV expression that have an impact on the behavior of these tumors. The study also screened 784 gliomas of various grades and histology and showed that *H3F3A* mutations were specific to GBM in children and young adults [33], again suggesting the importance of age distribution in reflecting tumor biology. Although this study identified the loss of function of a key component of the SWI/SNF complex, it did not analyze the expression of repetitive elements such as endogenous retroviral proteins.

To date, there have been only a few studies that have focused on the potential role of endogenous retroviruses in gliomas [15,147]. In a study of 300 glioma samples from the Chinese Glioma Genome Atlas (CGGA), the authors observed the aberrant presence of HERVK-113 in 90% of the tumors. This full-length polymorphic HERV-K is typically present in only 20% of the population and is capable of producing viral particles [155,156]; therefore, a disproportionate increase in the sampled tissue may implicate a contributory role in gliomagenesis. Recently, we found that a locus of *HML-6*, a heterogeneous but distinct family of elements in the HERV-K superfamily, is overexpressed in highly-invasive GBM cell lines and in patient-derived neurospheres; further, we demonstrated a decrease in survival in samples with elevated expression of the *HML-6* protein ERVK3-1 regardless of IDH mutation status [15]. Although the expression of SWI/SNF proteins was not examined in our study of GBM, it should be explored in any tumor with ERV expression in the future.

### 4.2. Embryonal Tumors

Embryonal tumors are primarily defined by sporadic recurrent genetic driver events and represent a heterogeneous group of tumors derived from embryonic brain tissue, which most commonly occur in infants under the age of three years and are histologically categorized by undifferentiated small round blue cells [157,158]. Embryonal tumors include AT/RT, medulloblastoma, neuroblastoma, cribriform neuroepithelial tumor (CRINET), and other rare entities [157,158]. In support of our hypothesis that dysfunction of chromatin remodeling pathways during development may lead to tumorigenesis through currently unrecognized pathways, it has recently been found that medulloblastoma tumors originate from a failure to eliminate a developmental cell population that expresses Sox2 and is derived from the external germinal layer [159]. There are multiple examples of embryonal tumors that contain LOF mutations in the SWI/SNF pathway, such as AT/RT, CRINET, and medulloblastoma; however, bi-allelic loss of *SMARCB1* in AT/RT has been the most well-characterized example [10,51,160,161,162].

We recently found that LOF of *SMARCB1*, a core protein in the SWI/SNF remodeling complex, in AT/RT leads to aberrant expression of the HERV-K (subtype HML-2) env protein, which results in the maintenance of pluripotency critical for tumorigenesis [10]. We found that 95% of AT/RT cases evaluated expressed the HERV-K envelope protein. We further observed expression of the envelope protein in both intracellular and extracellular compartments of four patient-derived AT/RT cell lines and observed an upregulation of HERV-K (HML-2) transcription when we knocked down expression of *SMARCB1* in neural stem cells [10]. We found that restoring *SMARCB1* expression to AT/RT cell lines led to a significant decrease in transcription of HML-2 loci and that INI1 (the protein encoded by *SMARCB1*) binds adjacent to the HML-2 promoter to repress its transcription in vitro in neural stem cells [10]. Like many other studies of HML-2 expression in tumors, we found that knocking down its expression resulted in decreased cell proliferation, cellular dispersion, and cell death in vitro [10,19,20]. Finally, we found that C-MYC was bound to the HML-2 promoter in the absence of SMARCB1 expression, and its binding resulted in increased HML-2 transcription [10]. Recently, it has also been shown that MYC can regulate multiple members of the SWI/SNF complex in tumors [163,164]. Due to the clear involvement of both SWI/SNF LOF and resultant ERV expression in AT/RT, it is likely this phenomenon may also occur in other embryonal tumors with *SMARCB1* or other SWI/SNF gene LOF, and these complex relationships should be further explored in future studies.

### 4.3. Cranial and Paraspinal Nerve Tumors

Nerve sheath tumors include both cranial and paraspinal nerve tumors, such as schwannoma, malignant peripheral nerve sheath tumor (MPNST), and neurofibroma, which originate from the neuroectoderm or the neural crest tissue in a developing embryo [165]. Schwannomas, which are normally benign WHO grade I tumors due to their low risk of metastatic spread and risk of recurrence after surgical resection [166], develop from Schwann cells, a later developmental stage of the shared precursor of these tumors, which typically encase and insulate nerves in the peripheral nervous system [165]. MPNSTs, which are frequently associated with either heritable or sporadic mutations in *NF1*, can result from prior radiation treatment and are malignant tumors with Schwann cell or perineural cell differentiation [3,165]. LOF of SWI/SNF proteins and/or significant alterations in their expression have been observed in both schwannomas and MPNST [62,63,64,65,161,167].

In a recent study of MPNST, the authors observed that 58% of MPNSTs evaluated (43/74) had aberrant ATRX expression with significantly less nuclear expression (<80%) than benign neurofibromas [65]. There was also an increase in aberrant ATRX expression in MPNST tumors with concurrent *NF1* mutations (65%) compared to sporadic MPNST tumors (48%) [65]. Patients with NF1-MPNST and aberrant expression of ATRX had a significantly decreased survival rate, and the authors suggest it could be used as a prognostic marker in this cancer [65]. In the epithelioid histopathologic subtype of MPNST, a rare tumor with diffuse S-100 positivity, infrequent association with *NF1*, and occasional malignant transformation from schwannoma, mutations in *SMARCB1* have also been observed in two-thirds of patients evaluated (n = 63) [62]. In another study, loss of *SMARCB1* expression was established by immunohistochemistry in 70% of patients; further, some cases of epithelioid MPNST (EMPNST) arose from pre-existing epithelioid schwannoma (ESCW), which also had a loss of *SMARCB1* expression in 40% of cases [63].

The timing of the acquisition of a *SMARCB1* mutation can have a major impact on which type of tumor develops, as evidenced by the initial discovery of *SMARCB1* mutations in both rhabdoid tumor predisposition syndrome and familial schwannomatosis [10,168,169,170]. Using mouse models to establish mutational timing effects, the authors displayed that *SMARCB1* loss in early neural crest cells was necessary to initiate tumorigenesis in the cranial nerves and meninges, leading to rhabdoid tumors, while *SMARCB1* loss with concomitant *NF2* LOF at a later developmental stage, in the Schwann cell lineage, led to a schwannoma [168]. These findings underscore the importance of the expression of tight control of developmental genes during CNS development and highlight the potential consequences of aberrant ERV and SWI/SNF expression in developing tissue. The continued expression of ERVs throughout and post-development, or their expression reactivation in terminally differentiated cells, could lead to different tumor types, especially in the absence of the Merlin protein, derived from *NF2* which is a known tumor suppressor gene [171]. In primary cells derived from Merlin-negative schwannoma and meningioma tumors, HERV-K was identified as a critical regulator of progression and a potential target for therapy [59].

### 4.4. Meningioma

Meningiomas comprise more than one-third of primary CNS tumors and are mostly comprised of slow-growing, benign WHO grade 1 tumors with heterogenous histologic features and a low risk of recurrence [172]. These tumors originate from arachnoid cap cells embryologically derived from neural crest cells and infrequently invade the brain or spinal cord [172]. Although meningiomas are normally considered benign, low-grade, and resectable tumors, there are subsets of these tumors that occur in difficult-to-access or eloquent locations, as well as subsets that have more malignant behavior and arise in very young individuals. In such cases, increasing the understanding of the interactions between epigenetic mechanisms and the control of ERV expression would greatly benefit our ability to develop targeted and less invasive treatments for these patients. In a case study of an untreated, rare intraventricular meningioma, whole-exome sequencing data revealed the presence of an *ARID1A* frameshift deletion in grade 2 and grade 3 sections of the tumor [173]. The patient also only had a single copy of chromosome 1p, indicating that the frameshift mutation present would lead to a sharp decrease in ARID1A protein [173]. *ARID1A* is the most frequently mutated member of the SWI/SNF complex and normally plays a role in enhancing BAF affinity to certain targets in chromatin as well as promoting the interactions between various proteins and nuclear hormone receptors to regulate gene expression and cell differentiation [174,175,176].

In another study of 255 meningiomas, including 63 WHO grade 1, 173 grade 2, and 19 grade 3 meningiomas, the authors found *ARID1A*, *SMARCA4*, and *SMARCB1* mutations in 17.3, 3.5, and 5.1% of samples, respectively [177]. *ARID1A* mutations occurred with similar frequency in both low- and high-grade meningiomas; however, mutations in *ARID1A* were found to be an independent poor prognostic indicator with a 7.421-fold increased hazard of death (*p* = 0.04) [177]. HERV-K expression has been observed in primary cells derived from patient meningiomas with *NF2* mutations; further, FDA-approved retroviral protease inhibitors such as ritonavir, atazanavir, and lopinavir reduced the proliferation of grade 1 meningioma cells [59]. In addition, there was one study that observed mutations in polybromo-1 (*PBRM1)* in both rhabdoid meningioma and papillary meningioma cases. *PRBM1* is also known as *RG1-associated factor 180* (*BAF180*), a member of the SWI/SNF complex, and is known to act as a tumor suppressor in many cancers, particularly in clear-cell renal cell carcinoma [67,178]. Both LOF of SWI/SNF proteins and HERV-K expression have been observed in meningioma samples; however, they have not yet been observed concomitantly in the same tumor sample. These tumors, especially those with SWI/SNF LOF, should be evaluated in a more robust manner and in a larger population to establish whether aberrant HERV-K expression and SWI/SNF alteration contribute towards mechanisms of tumorigenesis that also potentially serve as viable targets for treatment in these tumors.

## 5. Discussion

We have previously noticed, based on our translational experience, that some CNS tumors that are currently grouped together in the WHO classification schema do not exhibit uniform clinical behavior [5]. Based on these observations, we hypothesized that there may be additional biological criteria that could better stratify these tumors and explain our and others’ clinical observations. Further, based on our previous work in one such example of a rare CNS tumor with a high degree of heterogeneity and dichotomous clinical behavior, AT/RT, we believed that chromatin remodeling defects may further define these tumors and their relationship to endogenous retroviruses, which we have shown to be critical for tumorigenesis [10].

Based on the above, we were spurred to investigate the role of SWI/SNF mutations in a systematic fashion using WHO criteria [5]. We used the 2021 WHO classification schema as a guide for non-syndromic tumors of the CNS and correlated these tumors with SWI/SNF mutations and ERV involvement based on a systematic literature review. Further, we expanded our search to include related mutations as reported in the WHO classification, which we suspected could interact with the SWI/SNF complex as reported in the literature.

We found that approximately 20% of all tumors contained within the classification schema bear SWI/SNF LOF mutations. All the tumors in the WHO classification reported to have these LOF mutations may include both heritable first-hit mutations and sporadic mutations acquired later in life. We found that the rate of SWI/SNF LOF mutations in the tumors we evaluated aligns with the rate in general malignancy, and these mutations occur predominantly in tumor types that are not restricted by cell lineage, i.e., they occur before cells have been fated. Our observation of SWI/SNF defects and ERV expression in developmental tumors that affect young age groups, including those with bimodal age of distribution, supports our hypothesis about the timing of mutation acquisition in the SWI/SNF chromatin remodeling complex.

Most tumors with SWI/SNF mutations were high grade (75%) by the WHO grading system, grades III or IV, or they occurred in low-grade tumors exhibiting rare high-grade histopathologic features including anaplasia and cell de-differentiation [5]. Tumors with ERV expression followed many of the same themes as those with SWI/SNF LOF mutations across multiple cell lineages. While there is less evidence in the literature regarding ERVs in primary CNS tumors, there are multiple key examples of tumors with both SWI/SNF LOF mutations and ERV expression that can be used to elucidate the mechanisms of tumorigenesis that involve those pathways.

Thus far in our investigation, we have identified six primary CNS tumors across four categories of tumor types that bear both SWI/SNF LOF mutations and ERV involvement, and most of them (5/6 or 83.3%) are high grade by WHO criteria and/or feature a malignant phenotype by histopathology alone. The exception to this group is schwannomas, which are not typically considered malignant tumors and rarely harbor high-grade features. While schwannomas clinically and histopathologically do not appear consistent with the other malignant tumors in this category, they do in fact have SWI/SNF LOF and ERV expression. Further, the study of familial schwannomatosis was critical in the discovery of the role of SWI/SNF LOF in tumorigenesis. It is important to note that schwannomas are dynamic tumors that can transition to MPNST with different clinical characteristics and potentially a different underlying biology that may be more reminiscent of other malignant tumors in this same category [179]. It is possible that ERV expression plays a critical role in this malignant transformation and represents an interesting area for future investigation. In summary, there are several examples of CNS tumors with involvement of both LOF mutations in the SWI/SNF complex and ERV expression; however, we have identified many additional tumors with LOF of the SWI/SNF complex or ERV involvement that should be investigated in the future.

Our findings, guided by the WHO classification schema, have revealed that the current framework, which emphasizes the key importance of cell of origin, histopathologic description, and anatomic organization of the CNS, is an important foundation for understanding tumor behavior and clinical prognosis. However, the dichotomous biological and clinical behaviors of many CNS tumors suggest that our current framework is insufficient. Through epigenetic studies, we and other authors have found that CNS tumors currently grouped within the same categories may have different pathophysiologies based on epigenetic regulation that correlate more closely with clinical observations. Thus, epigenetic tools provide additional information that may be leveraged to better stratify these tumors. This may lead to better prognostication and intervention.

Epigenetic mechanisms contribute to heritable changes in expression that are not due to differences in underlying DNA sequences, and these pathways mediate the expression of many developmental processes, including the expression of endogenous retroviruses. We and others have shown that when ERV expression is not tightly regulated throughout and after development, aberrant expression can lead to tumorigenesis. However, this pathway has not been extensively explored in the context of SWI/SNF LOF in CNS tumors. We have shown the importance of intersectional investigations that focus on the global regulation of developmental genes and their contribution to cell differentiation, tumorigenesis, and the maintenance of pluripotency. Finally, the present expansion of tumor variants in the WHO CNS 5 includes many rare tumors with too few cases to render prognostications based on present knowledge of histopathology and molecular profiling, thereby reinforcing the importance of defining, understanding, and applying epigenetic mechanisms of tumorigenesis to tumor classification. The proposed relationship between mammalian SWI/SNF chromatin remodeling proteins and ERV elements represents one potential pathway for further investigation.

Additional pathways for better understanding these mechanisms and their potential contribution to tumorigenesis must also include consideration for alterations in genes or alternative epigenetic pathways with known targets involved in tumor development and that directly interact with the SWI/SNF chromatin remodeling complex, as such alterations likely influence their mechanism of action. Genes encoding transcription factors such as *Sox2* and *OCT4*, lineage-specific regulators such as *SHH*, *WNT*, and *NOTCH*, nuclear hormone receptors such as *GR* (glucocorticoid receptor), tumor suppressors such as *RB1*, and oncogenes such as *MYC* have been shown to interact with various subunits of the SWI/SNF complex [10,104,107,134,135,159,180]. For example, MYC binds to DNA sequences called E-boxes to recruit SWI/SNF subunits, as well as directly interacts with BRG1 to enhance its ability to remodel chromatin and activate gene expression [181]. Primary CNS tumors with MYC alterations include but are not limited to oligodendroglioma, adult and pediatric gliomas regardless of IDH status, central neurocytoma, spinal ependymoma, medulloblastoma, AT/RT, pineoblastoma, and diffuse large B-cell lymphoma [5]. Studies have also shown that important developmental signal transducers such as SHH directly interact with the SWI/SNF complex to regulate gene expression, specifically through the GLI family of transcription factors. Examples of tumors with this expression signature include medulloblastoma, choroid plexus carcinoma, AT/RT, and adamantinomatous craniopharyngioma [5]. MYC, TP53, and RB1 were the most frequent genetic mutations that have an established interaction with SWI/SNF in CNS tumors.

In addition to genes that interact with SWI/SNF, there are multiple epigenetic pathways that have a complex and dynamic relationship with chromatin remodeling. For example, the antagonistic relationship between the SWI/SNF complex and PRC is currently being exploited to develop therapeutic targets in SWI/SNF-deficient cancers. Alterations in PRC typically involve either direct mutations in core PRC2 subunits, such as EZH2, SUZ12, and EED, or point mutations in a histone site, such as H3K27M, which interacts with PRC2. These tumors include GBM, DMG, ependymoma, MPNST, anaplastic meningioma, and chondrosarcoma [5]. In addition to epigenetic mechanisms that involve direct histone modification and chromatin remodeling, the processing of small regulatory RNAs such as microRNAs involved in post-transcriptional regulation of gene expression also alters the SWI/SNF complex. One such example includes either the germline or sporadic mutation of DICER1, which is mediated by BAF155 to be recruited to genomic loci for processing precursor microRNAs into mature RNAs. CNS tumors with DICER1 mutations include pineoblastoma, primary intracranial sarcoma, and pituitary blastoma [5]. Notably, pineoblastoma and pituitary blastoma, which are recognized developmental tumors, occur in endocrine glands that are recognized as CNS border structures rather than true CNS parenchyma. Further, of the CNS tumors with non-SWI/SNF epigenetic alterations, several have documented aberrant ERV expression, such as Ewing’s sarcoma and primary B-cell lymphoma, both of which similarly arise from CNS border structures. Overall, this suggests to us that epigenetics may reveal a common theme in these tumors with dichotomous clinical behavior in these CNS border structures in that they may arise from cell lineages that have been developmentally arrested by the acquisition of defects in genetic mechanisms that are not inherited in Mendelian fashion. Further, as we have elaborated herein, many of these mechanisms may have common pathogenesis through the expression of ERVs. In addition, our review has revealed that many tumors with chromatin remodeling defects and ERV expression have overlapping histopathologic features such as syncytial appearance, which may reflect ERV biology and provide further support for stratification based on the existing WHO classification schema.

## 6. Conclusions and Future Directions

Our systematic review of the literature, guided by the most recent WHO CNS classification schema, has highlighted the need to marry our current framework based on histopathologic morphology with the insights evolved by the use of new technologies in epigenetic investigations. We have elaborated on trends between chromatin remodeling defects and expression of ERVs in tumors with a high degree of heterogeneity and dichotomous clinical behavior, suggesting that prognostication and intervention in these tumors may benefit from further translational stratification. In the future, we believe the field should focus on understanding the mechanistic relationship between ERVs and epigenetic pathways, with an emphasis on the SWI/SNF complex. This work may also potentially be applied to tumors outside of the CNS, which follow the concepts we have outlined above, particularly sinonasal cancers recently stratified by SMARCA4 mutations and primary tumors with SWI/SNF deficiencies that frequently metastasize to the CNS. We believe this review will be a useful resource in guiding the use of epigenetic tools in the next iteration of the WHO classification schema.

## Figures and Tables

**Table 1:** Primary central nervous system (CNS) tumors whose tumorigenesis is characterized by documented loss of function SWItch/sucrose non-fermentable (SWI/SNF) variants.

Primary CNS Tumors by WHO Classification Diagnosis	WHO Grade(s)	SWI/SNF Mutation(s)	References
**Gliomas, glioneuronal tumors, and neuronal tumors**			
*Adult-type diffuse gliomas*			
Astrocytoma, IDH-mutant	2–4	SMARCE1, SMARCA4, ATRX	[21,22,23,24,25,26]
Oligodendroglioma, IDH-mutant and 1p/19q-codeleted	2–3	BICRA (GLTSCR1); ARID1A	[22,27]
Glioblastoma, IDH-wildtype	4	ATRX	[28,29]
*Pediatric-type diffuse high-grade gliomas*			
Diffuse midline glioma, H3 K27-altered	4	SMARCA4; ATRX	[30,31]
Diffuse hemispheric glioma, H3 G34-mutant	4	ATRX	[32,33,34]
*Circumscribed astrocytic gliomas*			
Pilocytic astrocytoma	1	ATRX ^1^	[35]
High-grade astrocytoma with piloid features	3–4	ATRX	[35]
Pleomorphic xanthroastrocytoma	2–3	SMARCB1, ARID1A, ATRX	[36,37,38]
*Glioneuronal and neuronal tumors*			
Desmoplastic infantile ganglioglioma/desmoplastic infantile astrocytoma	1	ATRX ^1^	[39,40,41,42]
**Embryonal tumors**			
*Medulloblastoma*			
Medulloblastoma, WNT-activated	4	SMARCA4, SMARCB1, ARID1A, ARID2	[43]
Medulloblastoma, non-WNT/non-SHH (Group 3/4)	4	SMARCA4	[43,44]
*Other CNS embryonal tumors*			
Atypical teratoid/rhabdoid tumor	4	SMARCB1, SMARCA4	[45,46,47,48,49,50]
Cribriform neuroepithelial tumor	NR	SMARCB1	[51]
CNS tumor with BCOR internal tandem duplication	NR	SMARCA2	[52]
**Pineal tumors**			
Desmoplastic myxoid tumor of the pineal region, SMARCB1-mutant	NR	SMARCB1	[53]
**Cranial and paraspinal nerve tumors**			
Schwannoma	1	ARID1A, ARID1B; SMARCB1	[54,55,56,57,58,59]
Neurofibroma	1	SMARCA2	[60]
Malignant peripheral nerve sheath tumor	NR	SMARCB1; ATRX	[61,62,63,64,65,66]
**Meningioma**			
Clear cell meningioma	2	SMARCE1	[4,5]
Papillary meningioma	2–3	PBRM1	[59,67]
Rhabdoid meningioma	2–3	PBRM1	[67]
**Mesenchymal, non-meningothelial tumors involving the CNS**			
*Soft tissue tumors: tumors of uncertain differentiation*			
Primary intracranial sarcoma, DICER1-mutant	NR	ATRX	[68]
*Notochordal tumors*			
Chordoma	NR	SMARCB1	[69,70]
**Germ cell tumors**			
Immature teratoma	NR	SMARCA4	[71]

^1^ Reported in tumors with anaplastic features or recurrent cases with glioblastoma-like histology only. *Abbreviations: ARID1A (AT-rich interaction domain 1A); ARID1B (AT-rich interaction domain 1B); ARID2 (AT-rich interaction domain 2); ATRX (alpha-thalassemia/mental retardation, X-linked); BCOR (BCL6 corepressor); BICRA (BRD4 interacting chromatin remodeling complex associated protein); GLTSCR1 (glioma tumor suppressor candidate region gene 1); H3K27 (histone 3 on lysine 27); H3K36 (histone 3 on lysine 36); H3G34 (histone 3 on arginine 34); IDH (isocitrate dehydrogenase); NR (not reported); PBRM1 (polybromo-1); SHH (sonic hedgehog); SMARCA2 (SWI/SNF-related matrix-associated, actin-dependent regulator of chromatin subfamily A, member 2); SMARCA4 (SWI/SNF-related matrix-associated, actin-dependent regulator of chromatin subfamily A, member 4); SMARCB1 (SWI/SNF-related matrix-associated, actin-dependent regulator of chromatin subfamily B, member 1); SMARCE1 (SWI/SNF-related matrix-associated, actin-dependent regulator of chromatin subfamily E, member 1); WNT (wingless-related integration site)*.

**Table 2:** Primary CNS tumors whose tumorigenesis is characterized by documented endogenous retroviral (ERV) expression.

Primary CNS Tumors by WHO Classification Diagnosis	WHO Grade(s)	HERV(s) Expressed	Non-Human ERV(s) Expressed	References
**Gliomas, glioneuronal tumors, and neuronal tumors**				
*Adult-type diffuse gliomas*				
Glioblastoma, IDH-wildtype	4	HERV1, HERVK, HERVL, ERV3, HML-6 (ERVK3-1)	-	[78,79]
**Choroid plexus tumors**				
Choroid plexus papilloma	1	-	Rous sarcoma virus	[72]
**Embryonal tumors**				
*Other CNS embryonal tumors*				
Atypical teratoid/rhabdoid tumor	4	HERV-K	-	[10]
**Cranial and paraspinal nerve tumors**				
Schwannoma	1	HERV-K	-	[59]
Perineuroma	1	-	ALV	[74,76]
Malignant peripheral nerve sheath tumor	NR	-	ALV	[66]
**Meningioma**				
Meningothelial meningioma	1–3	HERV-K ^1^	-	[59]
Fibrous meningioma	1–3	HERV-K ^1^	-	[59]
Transitional meningioma	1–3	HERV-K ^1^	-	[59]
Psammomatous meningioma	1–3	HERV-K ^1^	-	[59]
Angiomatous meningioma	1–3	HERV-K ^1^	-	[59]
Microcystic meningioma	1–3	HERV-K ^1^	-	[59]
Secretory meningioma	1–3	HERV-K ^1^	-	[59]
Lymphoplasmacyte-rich meningioma	1–3	HERV-K ^1^	-	[59]
Metaplastic meningioma	1–3	HERV-K ^1^	-	[59]
Chordoid meningioma	2	HERV-K ^1^	-	[59]
Clear cell meningioma	2	HERV-K ^1^	-	[59]
Papillary meningioma	2–3	HERV-K ^1^	-	[59,67]
Rhabdoid meningioma	2–3	HERV-K ^1^	-	[59,67]
Atypical meningioma	2–3	HERV-K ^1^	-	[59]
Anaplastic (malignant) meningioma	3	HERV-K ^1^	-	[59]
**Mesenchymal, non-meningothelial tumors involving the CNS**				
*Soft tissue tumors: vascular tumors*				
Hemangiomas and vascular malformations	NR	-	ALV	[75]
*Soft tissue tumors: skeletal muscle tumors*				
Rhabdomyosarcoma	N/A	ERV-9	Feline endogenous retrovirus	[77]
*Soft tissue tumors: tumors of uncertain differentiation*				
Ewing sarcoma	4	Syncytin-1, ERV-L	-	[80]
**Melanocytic tumors**				
*Circumscribed meningeal melanocytic neoplasms*				
Melanocytoma and melanoma	NR	-	MMVL30	[81]
**Hematolymphoid tumors involving the CNS**				
*Lymphomas: CNS lymphomas*				
Primary diffuse large B-cell lymphoma of the CNS	NR	Variety— Unspecified	-	[82]
*Histiocytic tumors*				
Langerhans cell histiocytosis	NR	-	Primate type D retroviruses, murine intracisternal A particles, Jaagsiekte sheep retrovirus, and murine long interspersed nuclear elements	[73]
**Germ cell tumors**				
Mature teratoma	NR	ERVK24	-	[83]
Germinoma	NR	ERVK24	-	[83]
Embryonal carcinoma	NR	ERVK24	-	[83]
Yolk sac tumor	NR	ERVK24	-	[83]
Choriocarcinoma	NR	ERVK24	-	[83]

^1^ Reported in meningiomas without concomitant Merlin mutation. *Abbreviations: ALV (avian leukosis virus); ERV (endogenous retrovirus); ERV3 (endogenous retrovirus group 3); ERV9 (endogenous retrovirus group 9); ERVK24 (endogenous retrovirus group K member 24); ERVK3-1 (endogenous retrovirus group 3 member 1); HERV1 (human endogenous retrovirus group 1); HERVK (human endogenous retrovirus group K); HERVL (human endogenous retrovirus group L); HML-6 (human endogenous MMTV-like 6); MMVL30 (mouse murine leukemia virus group L member 30)*.

**Table 3:** Primary CNS tumors whose tumorigenesis is characterized by both LOF SWI/SNF mutations and aberrant ERV expression.

Primary CNS Tumors by WHO Classification Diagnosis	WHO Grade(s)	SWI/SNF Mutation(s)	ERV(s) Expressed	References
**Gliomas, glioneuronal tumors, and neuronal tumors**				
*Adult-type diffuse gliomas*				
Glioblastoma, IDH-wildtype	4	ATRX	HERV1, HERVK, HERVL, ERV3, HML-6 (ERVK3-1)	[28,29,78,79]
**Embryonal tumors**				
*Other CNS embryonal tumors*				
Atypical teratoid/rhabdoid tumor	4	SMARCB1, SMARCA4	HERV-K, Syncytin-1, Syncytin-2	[45,46,47,48,49,50]
**Cranial and paraspinal nerve tumors**				
Schwannoma	1	ARID1A, ARID1B; SMARCB1	HERV-K	[54,55,56,57,58,59]
Malignant peripheral nerve sheath tumor	NR	SMARCB1; ATRX	ALV	[61,62,63,64,65,66]
**Meningioma (Merlin(-))**				
Papillary meningioma	2–3	PBRM1	HERV-K ^1^	[59,67]
Rhabdoid meningioma	2–3	PBRM1	HERV-K ^1^	[59,67]

^1^ Also reported in a meningioma without concomitant Merlin mutation.

**Table 4:** Primary CNS tumors whose tumorigenesis is characterized by alternative genetic and/or epigenetic pathways with known direct documented interaction with SWI/SNF subunits.

Primary CNS Tumors by WHO Classification Diagnosis	WHO Grade(s)	Pathways Interacting with SWI/SNF	References
**Gliomas, glioneuronal tumors, and neuronal tumors**			
*Adult-type diffuse gliomas*			
Astrocytoma, IDH-mutant	2–4	TP53, MYC, RB1	[84]
Oligodendroglioma, IDH-mutant and 1p/19q-codeleted	2–3	SETD2, MYC, RB1	[85,86,87]
Glioblastoma, IDH-wildtype	4	EZHIP, SETD2, TP53, MYC, RB1	[28,88]
*Pediatric-type diffuse low-grade gliomas*			
Polymorphous low-grade neuroepithelial tumor of the young	1	TP53 ^1^, RB1 ^1^	[89]
*Pediatric-type diffuse high-grade gliomas*			
Diffuse midline glioma, H3 K27-altered	4	EZHIP, H3K27, TP53	[4,5,90,91]
Diffuse hemispheric glioma, H3 G34-mutant	4	H3G34, TP53, MYCN	[4,5,92]
Diffuse pediatric-type high-grade glioma, H3-wildtype, and IDH-wildtype	4	TP53, MYCN	[93]
*Circumscribed astrocytic gliomas*			
Pleomorphic xanthroastrocytoma	2–3	TP53 ^2^, NOTCH ^2^	[37]
Astroblastoma, MN1-altered	NR	MN1	[94]
Glioneuronal and neuronal tumors			
Desmoplastic infantile ganglioglioma/desmoplastic infantile astrocytoma	1	TP53	[39,40,41,42]
Central neurocytoma	2	MYCN	[95]
Cerebellar liponeurocytoma	2	TP53	[96]
*Ependymal tumors*			
Supratentorial ependymoma, ZFTA fusion-positive	2–3	ZFTA-RELA	[97]
Supratentorial ependymoma, YAP1 fusion-positive	2–3	YAP1	[98]
Posterior fossa ependymoma	2–3	EZHIP	[99]
Posterior fossa ependymoma (Group A)	2–3	EZHIP	[99]
Spinal ependymoma, MYCN-amplified	2–3	MYCN	[100,101,102,103]
**Choroid plexus tumors**			
Choroid plexus papilloma	1	TP53 ^2^	[104]
Choroid plexus carcinoma	3	TP53, SHH, NOTCH	[104,105,106]
**Embryonal tumors**			
*Medulloblastoma*			
Medulloblastoma, WNT-activated	4	WNT/CTNNB1, TP53, OCT4	[4,5,107]
Medulloblastoma, SHH-activated and TP53-wildtype	4	SHH, CREBBP, MYCN, MYCL, YAP1, OCT4	[4,5,43,107,108,109]
Medulloblastoma, SHH-activated and TP53-mutant	4	TP53, SHH, CREBBP, OCT4	[4,5,107,108,109]
Medulloblastoma, non-WNT/non-SHH (Group 3/4)	4	MYC	[110]
*Other CNS embryonal tumors*			
Atypical teratoid/rhabdoid tumor	4	SHH, NRAS, MYC	[4,5]
Embryonal tumor with multilayered rosettes	4	LIN28A; TP53	[4,5,111]
**Pineal tumors**			
Pineoblastoma	4	DICER1; RB1, MYC	[112,113,114]
**Cranial and paraspinal nerve tumors**			
Malignant peripheral nerve sheath tumor	NR	SUZ12, EED, H3K27, TP53	[4,5,115,116,117]
**Meningioma**			
Secretory meningioma	1–3	KLF4	[118]
Atypical meningioma	2–3	TP53	[5,119]
Anaplastic (malignant) meningioma	3	H3K27, TP53	[4,5,119]
**Mesenchymal, non-meningothelial tumors involving the CNS**			
*Soft tissue tumors: fibroblastic and myofibroblastic tumors*			
Solitary fibrous tumor	1–3	EP300, TP53	[5,120,121,122]
*Soft tissue tumors: tumors of uncertain differentiation*			
Intracranial mesenchymal tumor, FET::CREB fusion-positive	N/A	FET/CREBBP	[123]
CIC-rearranged sarcoma	4	NUTM1	[4,5]
Primary intracranial sarcoma, DICER1-mutant	NR	DICER1; TP53	[5,112]
Ewing sarcoma	4	FET; TP53	[5,123,124,125,126]
*Chondro-osseous tumors: chondrogenic tumors*			
Mesenchymal chondrosarcoma	NR	NOTCH, NCoA-2	[127]
Chondrosarcoma	1–3	RB1, H3K36	[128,129,130]
**Melanocytic tumors**			
*Diffuse meningeal melanocytic neoplasms*			
Melanocytosis and melanomatosis	NR	YAP1	[131]
*Circumscribed meningeal melanocytic neoplasms*			
Melanocytoma and melanoma	NR	YAP1	[131]
**Hematolymphoid tumors involving the CNS**			
*Lymphomas: CNS lymphomas*			
Primary diffuse large B-cell lymphoma of the CNS	NR	MYC	[132]
*Lymphomas: miscellaneous rare lymphomas in the CNS*			
MALT lymphoma of the dura	NR	NOTCH	[4,5]
*Histiocytic tumors*			
Erdheim–Chester disease	NR	NRAS	[133]
**Germ cell tumors**			
Mature teratoma	NR	JMJD1C, RB1	[4,5,134,135]
Immature teratoma	NR	JMJD1C, RB1	[4,5,134,135]
Teratoma with somatic-type malignancy	NR	JMJD1C	[4,5,134]
Germinoma	NR	JMJD1C, LIN28A, TP53, RB1	[4,5,134,135,136]
Embryonal carcinoma	NR	JMJD1C, LIN28A, RB1	[4,5,134,135]
Yolk sac tumor	NR	JMJD1C, LIN28A, RB1; TP53	[4,5,134,135,136]
Choriocarcinoma	NR	JMJD1C, RB1	[4,5,134,135]
Mixed germ cell tumor	NR	JMJD1C, RB1	[4,5,134,135]
**Tumors of the sellar region**			
Adamantinomatous craniopharyngioma	1	SHH, CTNNB1	[137]
Pituitary adenoma/pituitary neuroendocrine tumor	NR	Ik1, Ik2, TP53	[138,139]
Pituitary blastoma	NR	DICER1, TP53	[4,5,140,141,142]

^1^ Reported in one recurrent case with glioblastoma-like histology. ^2^ Reported in rare tumors with anaplastic features only. *Abbreviations: CREBBP (cAMP-response element binding protein); CTNNB1 (catenin beta-1); EED (embryonic ectoderm development); EP300 (E1A-associated protein p300); EZHIP (enhancer of zeste homologs inhibitory protein); Ik1 (Ikaros 1); Ik2 (Ikaros 2); JMJD1C (Jumonji domain containing 1C); KLF4 (Kruppel-like factor 4); LIN28A (Lin-28 homolog A); MALT (mucosa-associated lymphoid tissue); MN1 (meningioma (disrupted in balanced translocation) 1); NCoA-2 (nuclear receptor coactivator 2); NOTCH (neurogenic locus notch homolog protein); NRAS (neuroblastoma ras viral oncogene homolog); NUTM1 (NUT midline carcinoma family member 1); OCT4 (octamer-binding transcription 4); RB1 (retinoblastoma transcriptional corepressor 1); RELA (V-rel reticuloendotheliosis viral oncogene homolog A); SETD2 (SET domain-containing 2); SUZ12 (zeste 12 homolog); TP53 (tumor protein 53); YAP1 (yes-associated protein-1); ZFTA (zinc finger translocation associated)*.

**Table 5:** Primary CNS tumors with no known documented loss-of-function (LOF) SWI/SNF mutations, genetic/epigenetic variants that directly interact with the mammalian SWI/SNF complex, or harbor known aberrant ERV expression investigated to date.

Primary CNS Tumors by WHO Classification Diagnosis	WHO Grade(s)	SWI/SNF Mutation(s)	Other Epigenetic Pathways Implicated	Human ERV(s)	Non-Human ERV(s)
**Gliomas, glioneuronal tumors, and neuronal tumors**					
*Pediatric-type diffuse low-grade gliomas*					
Diffuse astrocytoma, MYB-or MYBL1-altered	1	N/A	N/A	N/A	N/A
Angiocentric glioma	1	N/A	N/A	N/A	N/A
Polymorphous low-grade neuroepithelial tumor of the young	1	N/A	TP53 ^1^; RB1 ^1^	N/A	N/A
Diffuse low-grade glioma, MAPK pathway-altered	1	N/A	N/A	N/A	N/A
*Pediatric-type diffuse high-grade gliomas*					
Infant-type hemispheric glioma	NR	N/A	N/A	N/A	N/A
*Circumscribed astrocytic gliomas*					
Pilocytic astrocytoma	1	ATRX ^2^	N/A	N/A	N/A
Subependymal giant cell astrocytoma	1	N/A	N/A	N/A	N/A
Chordoid glioma	2	N/A	N/A	N/A	N/A
*Glioneuronal and neuronal tumors*					
Ganglioglioma	1	N/A	N/A	N/A	N/A
Gangliocytoma	1	N/A	N/A	N/A	N/A
Desmoplastic infantile ganglioglioma/desmoplastic infantile astrocytoma	1	ATRX ^2^	TP53 ^2^	N/A	N/A
Dysembryoplastic neuroepithelial tumor	1	N/A	N/A	N/A	N/A
Diffuse glioneuronal tumor with oligodendroglioma-like features and nuclear clusters	NR	N/A	N/A	N/A	N/A
Papillary glioneuronal tumor	1	N/A	N/A	N/A	N/A
Rosette-forming glioneuronal tumor	1	N/A	N/A	N/A	N/A
Myxoid glioneuronal tumor	1	N/A	N/A	N/A	N/A
Diffuse leptomeningeal glio- neuronal tumor	NR	N/A	N/A	N/A	N/A
Multinodular and vacuolating neuronal tumor	1	N/A	N/A	N/A	N/A
Dysplastic cerebellar gangliocytoma (Lhermitte-Duclos disease)	1	N/A	N/A	N/A	N/A
Extraventricular neurocytoma	2	N/A	N/A	N/A	N/A
*Ependymal tumors*					
Supratentorial ependymoma (fusion-negative)	2–3	N/A	N/A	N/A	N/A
Posterior fossa group B (PFB) ependymoma	2–3	N/A	N/A	N/A	N/A
Spinal ependymoma	2–3	N/A	N/A	N/A	N/A
Myxopapillary ependymoma	2	N/A	N/A	N/A	N/A
Subependymoma	1	N/A	N/A	N/A	N/A
**Choroid plexus tumors**					
Atypical choroid plexus papilloma	2	N/A	N/A	N/A	N/A
**Embryonal tumors**					
*Other CNS embryonal tumors*					
CNS neuroblastoma, FOXR2- activated	4	N/A	N/A	N/A	N/A
CNS embryonal tumor NEC/NOS	3–4	N/A	N/A	N/A	N/A
**Pineal tumors**					
Pineocytoma	1	N/A	N/A	N/A	N/A
Pineal parenchymal tumor of intermediate differentiation	2–3	N/A	N/A	N/A	N/A
Papillary tumor of the pineal region	2–3	N/A	N/A	N/A	N/A
**Cranial and paraspinal nerve tumors**					
Hybrid nerve sheath tumors ^3^	NR	N/A	N/A	N/A	N/A
Malignant melanocytic nerve sheath tumor	NR	N/A	N/A	N/A	N/A
Cauda equina neuroendocrine tumor (previously paraganglioma)	1	N/A	N/A	N/A	N/A
**Mesenchymal, non-meningothelial tumors involving the CNS**					
*Soft tissue tumors: vascular tumors*					
Hemangioblastoma	1	N/A	N/A	N/A	N/A
**Hematolymphoid tumors involving the CNS**					
*Lymphomas: CNS lymphomas*					
Immunodeficiency-associated CNS lymphomas	NR	N/A	N/A	N/A	N/A
Lymphomatoid granulomatosis	1–3	N/A	N/A	N/A	N/A
Intravascular large B-cell lymphoma	NR	N/A	N/A	N/A	N/A
*Lymphomas: miscellaneous rare lymphomas in the CNS*					
Other low-grade B-cell lymphomas of the CNS	NR	N/A	N/A	N/A	N/A
Anaplastic large cell lymphoma (ALK+/ALK−)	NR	N/A	N/A	N/A	N/A
T-cell and NK/T-cell lymphomas	NR	N/A	N/A	N/A	N/A
*Histiocytic tumors*					
Rosai–Dorfman disease	NR	N/A	N/A	N/A	N/A
Juvenile xanthogranuloma	NR	N/A	N/A	N/A	N/A
Histiocytic sarcoma	NR	N/A	N/A	N/A	N/A
**Tumors of the sellar region**					
Papillary craniopharyngioma	1	N/A	N/A	N/A	N/A
Pituicytoma, granular cell tumor of the sellar region, spindle cell oncocytoma	NR	N/A	N/A	N/A	N/A

^1^ Reported in only one recurrent case with glioblastoma-like histopathology [89].^2^ Reported in atypical tumors with anaplastic features only [35]. ^3^ Dependent on composition of tumor subtypes contained within primary tumor. *Abbreviations: ALK (anaplastic lymphoma kinase); FOXR2 (forkhead box R2); MAPK (mitogen-activated protein kinase); MYBL1 (MYB proto-oncogene like 1); N/A (not applicable); NEC (not elsewhere classified); NK (natural killer); NOS (not otherwise specified).*

## Data Availability

The data used to generate original datasets in this study (Table 1, Table 2, Table 3, Table 4 and Table 5, Supplemental Tables S1 and S2) is publicly available within the 5th edition of the World Health Organization Classification of Tumours of the Central Nervous System.

## Supplementary Materials

The following supporting information can be downloaded at: https://www.mdpi.com/article/10.3390/cancers15092511/s1.
