# Supplementary material for: Epigenetic Regulation in Primary CNS Tumors: An Opportunity to Bridge Old and New WHO Classifications
Source: Cancers (Basel). 2023 Apr 27;15(9):2511. doi: 10.3390/cancers15092511 (PMC10177493; doi:10.3390/cancers15092511)
Supplement: Supplementary file 1 [file cancers-15-02511-s001.zip › cancers-2258129-supplementary.pdf]

SUPPLEMENTAL FIGURES

Primary CNS Tumors by WHO Classification Diagnosis	WHO Grade(s)	SWI/SNF Mutation(s)	Pathways Interacting with SWI/SNF	Human ERV(s) Expressed	Non-Human ERV(s) Expressed	References
**Gliomas, glioneuronal tumors, and neuronal tumors**						
Adult-type diffuse gliomas						
Astrocytoma, IDH-mutant	2-4	SMARCE1, SMARCA4, ATRX	TP53, MYC, RB1	-	-	[5,21–26,84]
Oligodendroglioma, IDH-mutant and 1p/19q-codeleted	2-3	BICRA (GLTSCR1); ARID1A	SETD2, MYC; RB1	-	-	[5,22,27,85–87]
Glioblastoma, IDH-wildtype	4	ATRX	EZHIP, SETD2; TP53; MYC; RB1	HERV1, HERVK, HERVL, ERV3, HML-6 (ERVK3-1)	-	[5,28,29,78,79,88]
Pediatric-type diffuse low-grade gliomas						
Diffuse astrocytoma, MYB- or MYBL1-altered	1	-	-	-	-	
Angiocentric glioma	1	-	-	-	-	
Polymorphous low-grade neuroepithelial tumour of the young	1	-	TP53/RB1[1]	-	-	[5,89]
Diffuse low-grade glioma, MAPK pathway-altered	1	-	-	-	-	
Pediatric-type diffuse high-grade gliomas						
Diffuse midline glioma, H3 K27-altered	4	SMARCA4; ATRX	EZHIP, H3K27; TP53	-	-	[5,30,31,90,91]
Diffuse hemispheric glioma, H3 G34-mutant	4	ATRX	H3G34; TP53; MYCN	-	-	[5,32–34,92]
Diffuse pediatric-type high-grade glioma, H3-wildtype and IDH-wildtype	4	-	TP53, MYCN	-	-	[5,93]
Infant-type hemispheric glioma	NR	-	-	-	-	
Circumscribed astrocytic gliomas						
Pilocytic astrocytoma	1	ATRX[2]	-	-	-	[35]
High-grade astrocytoma with piloid features	3-4	ATRX	-	-	-	[35]
Pleomorphic xanthroastrocytoma	2-3	SMARCB1, ARID1A, ATRX	TP53[2], NOTCH[2]	-	-	[5,36–38]
Subependymal giant cell astrocytoma	1	-	-	-	-	
Chordoid glioma	2	-	-	-	-	

Astroblastoma, MN1-altered	NR	-	MN1	-	-	[94]
Glioneuronal and neuronal tumors						
Ganglioglioma	1	-	-	-	-	
Gangliocytoma	1	-	-	-	-	
Desmoplastic infantile ganglioglioma/desmoplastic infantile astrocytoma	1	ATRX[1]	TP53[1]	-	-	[5,39–42]
Dysembryoplastic neuroepithelial tumor	1	-	-	-	-	
Diffuse glioneuronal tumor with oligodendroglioma-like features and nuclear clusters	NR	-	-	-	-	
Papillary glioneuronal tumor	1	-	-	-	-	
Rosette-forming glioneuronal tumor	1	-	-	-	-	
Myxoid glioneuronal tumor	1	-	-	-	-	
Diffuse leptomeningeal glioneuronal tumor	NR	-	-	-	-	
Multinodular and vacuolating neuronal tumor	1	-	-	-	-	
Dysplastic cerebellar gangliocytoma (Lhermitte-Duclos disease)	1	-	-	-	-	
Central neurocytoma	2	-	MYCN	-	-	[5,95]
Extraventricular neurocytoma	2	-	-	-	-	
Cerebellar liponeurocytoma	2	-	TP53	-	-	[5,96]
Ependymal tumors						
Supratentorial ependymoma (fusion-negative)	2-3	-	-	-	-	
Supratentorial ependymoma, ZFTA fusion-positive	2-3	-	ZFTA-RELA	-	-	[97]
Supratentorial ependymoma, YAP1 fusion-positive	2-3	-	YAP1	-	-	[98]
Posterior fossa ependymoma	2-3	-	EZHIP	-	-	[99]
Posterior fossa group A (PFA) ependymoma	2-3	-	EZHIP	-	-	[99]
Posterior fossa group B (PFB) ependymoma	2-3	-	-	-	-	
Spinal ependymoma	2-3	-	-	-	-	

Spinal ependymoma, MYCN-amplified	2-3	-	MYCN	-	-	[5,100–103]
Myxopapillary ependymoma	2	-	-	-	-	
Subependymoma	1	-	-	-	-	
**Choroid plexus tumors**						
Choroid plexus papilloma	1	-	TP53 (rare)	-	Roux Sarcoma Virus	[5,72,104]
Atypical choroid plexus papilloma	2	-	-	-	-	
Choroid plexus carcinoma	3	-	TP53; SHH, NOTCH	-	-	[5,104–106]
**Embryonal tumors**						
Medulloblastoma						
Medulloblastoma, WNT-activated	4	SMARCA4, SMARCB1, ARID1A, ARID2	WNT/CTNNB1, TP53, OCT4	-	-	[5,43,107]
Medulloblastoma, SHH-activated and TP53-wildtype	4	-	SHH, CREBBP; MYCN, MYCL, YAP1, OCT4	-	-	[5,43,44,107,109]
Medulloblastoma, SHH-activated and TP53-mutant	4	-	TP53, SHH, CREBBP, OCT4	-	-	[5,44,107,109]
Medulloblastoma, non-WNT/non-SHH (Group 3/4)	4	SMARCA4	MYC	-	-	[5,107,109,110]
Medulloblastoma, histologically defined	4	Not relevant	Not relevant	-	-	
Other CNS embryonal tumors						
Atypical teratoid/rhabdoid tumor	4	SMARCB1, SMARCA4	SHH, NRAS, MYC	HERV-K	-	[5,10,45–50]
Cribriform neuroepithelial tumor	NR	SMARCB1	-	-	-	[51]
Embryonal tumor with multilayered rosettes	4	-	LIN28A; TP53	-	-	[5,111]
CNS neuroblastoma, FOXR2-activated	4	-	-	-	-	
CNS tumor with BCOR internal tandem duplication	NR	SMARCA2	-	-	-	[52]
CNS embryonal tumor NEC/NOS	3-4	-	-	-	-	
**Pineal tumors**						
Pineocytoma	1	-	-	-	-	
Pineal parenchymal tumor of intermediate differentiation	2-3	-	-	-	-	

Pineoblastoma	4	-	DICER1; RB1; MYC	-	-	[112–114]
Papillary tumor of the pineal region	2-3	-	-	-	-	
Desmoplastic myxoid tumor of the pineal region, SMARCB1-mutant	NR	SMARCB1	-	-	-	[53]
**Cranial and paraspinal nerve tumors**						
Schwannoma	1	ARID1A, ARID1B; SMARCB1	-	HERV-K	-	[54–59]
Neurofibroma	1	SMARCA2	-	-	-	[60]
Perineuroma	1	-	-	-	ALV	[74,76]
Hybrid nerve sheath tumors[3]	NR	-	-	-	-	
Malignant melanocytic nerve sheath tumor	NR	-	-	-	-	
Malignant peripheral nerve sheath tumor	NR	SMARCB1; ATRX	SUZ12, EED, H3K27; TP53	ALV	ALV	[61–66,115–117]
Cauda equina neuroendocrine tumor (previously paraganglioma)	1	-	-	-	-	
**Meningioma**						
Meningothelial meningioma	1-3	-	-	HERV-K[4]	-	[59]
Fibrous meningioma	1-3	-	-	HERV-K[4]	-	[59]
Transitional meningioma	1-3	-	-	HERV-K[4]	-	[59]
Psammomatous meningioma	1-3	-	-	HERV-K[4]	-	[59]
Angiomatous meningioma	1-3	-	-	HERV-K[4]	-	[59]
Microcystic meningioma	1-3	-	-	HERV-K[4]	-	[59]
Secretory meningioma	1-3	-	KLF4	HERV-K[4]	-	[59,118]
Lymphoplasmacyte-rich meningioma	1-3	-	-	HERV-K[4]	-	[59]
Metaplastic meningioma	1-3	-	-	HERV-K[4]	-	[59]
Chordoid meningioma	2	-	-	HERV-K[4]	-	[59]
Clear cell meningioma	2	SMARCE1	-	HERV-K[4]	-	[59]
Papillary meningioma	2-3	PBRM1	-	HERV-K[4]	-	[59,67]
Rhabdoid meningioma	2-3	PBRM1	-	HERV-K[4]	-	[59,67]
Atypical meningioma	2-3	-	TP53	HERV-K[4]	-	[59,119]
Anaplastic (malignant) meningioma	3	-	H3K27M; TP53	HERV-K[4]	-	[5,59,119]
**Mesenchymal, non-meningothelial tumors involving the CNS**						
Soft tissue tumors: Fibroblastic and myofibroblastic tumors						

Solitary fibrous tumor	1-3	-	EP300; TP53	-	-	[120–122]
Soft tissue tumors: Vascular tumors						
Hemangiomas and vascular malformations	NR	-	-	-	ALV	[75]
Hemangioblastoma	1	-	-	-	-	
Soft tissue tumors: Skeletal muscle tumors						
Rhabdomyosarcoma	NR	-	-	ERV-9	Feline endogenous retrovirus	[77]
Soft tissue tumors: Tumors of uncertain differentiation						
Intracranial mesenchymal tumor, FET::CREB fusion-positive	NR	-	FET	-	-	[123]
CIC-rearranged sarcoma	4	-	NUTM1	-	-	[5]
Primary intracranial sarcoma, DICER1-mutant	NR	ATRX	DICER1; TP53	-	-	[68,112]
Ewing sarcoma	4	-	FET; TP53	Syncytin-1; ERV-L	-	[80,123–126]
Chondro-osseous tumors: Chondrogenic tumors						
Mesenchymal chondrosarcoma	NR	-	NOTCH, NCoA-2	-	-	[127]
Chondrosarcoma	1-3	-	RB1, H3K36	-	-	[128-130]
Notochordal tumors						
Chordoma	NR	SMARCB1	-	-	-	[69,70]
**Melanocytic tumors**						
Diffuse meningeal melanocytic neoplasms						
Melanocytosis and melanomatosis	NR	-	YAP1	-	-	[131]
Circumscribed meningeal melanocytic neoplasms						
Melanocytoma and melanoma	NR	-	YAP1	-	MMVL30	[81,131]
**Hematolymphoid tumors involving the CNS**						
Lymphomas: CNS lymphomas						
Primary diffuse large B-cell lymphoma of the CNS	NR	-	MYC	Variety - Unspecified	-	[82,132]
Immunodeficiency-associated CNS lymphomas	NR	-	-	-	-	
Lymphomatoid granulomatosis	1-3	-	-	-	-	
Intravascular large B-cell lymphoma	NR	-	-	-	-	
Lymphomas: Miscellaneous rare lymphomas in the CNS						

MALT lymphoma of the dura	NR	-	NOTCH	-	-	[5]
Other low-grade B-cell lymphomas of the CNS	NR	-	-	-	-	
Anaplastic large cell lymphoma (ALK+/ALK-)	NR	-	-	-	-	
T-cell and NK/T-cell lymphomas	NR	-	-	-	-	
Histiocytic tumors						
Erdheim-Chester disease	NR	-	NRAS	-	-	[133]
Rosai-Dorfman disease	NR	-	-	-	-	
Juvenile xanthogranuloma	NR	-	-	-	-	
Langerhans cell histiocytosis	NR	-	-	-	primate type D retroviruses, murine intracisternal A particles, Jaagsiekte sheep retrovirus, and murine long interspersed nuclear elements	[73]
Histiocytic sarcoma	NR	-	-	-	-	
**Germ cell tumors**						
Mature teratoma	NR	-	JMJD1C; RB1	ERVK24	-	[83,134,135]
Immature teratoma	NR	SMARCA4	JMJD1C; RB1	-	-	[71,134,135]
Teratoma with somatic-type malignancy	NR	-	JMJD1C	-	-	[134]
Germinoma	NR	-	JMJD1C, LIN28A; TP53; RB1	ERVK24	-	[83,134–136]
Embryonal carcinoma	NR	-	JMJD1C, LIN28A; RB1	ERVK24	-	[83,134,135]
Yolk sac tumor	NR	-	JMJD1C, LIN28A; TP53; RB1	ERVK24	-	[83,134-136]
Choriocarcinoma	NR	-	JMJD1C; RB1	ERVK24	-	[83,134,135]
Mixed germ cell tumor[3]	NR	-	JMJD1C; RB1	-	-	[134,135]
**Tumors of the sellar region**						
Adamantinomatous craniopharyngioma	1	-	SHH, CTNNB1 (B-catenin)	-	-	[137]
Papillary craniopharyngioma	1	-	-	-	-	

Pituicytoma, Granular cell tumor of the sellar region, Spindle cell oncocytoma	NR	-	-	-	-	
Pituitary adenoma/pituitary neuroendocrine tumor	NR	-	Ik1, Ik2; TP53[5]	-	-	[138,139]
Pituitary blastoma	NR	-	DICER1; TP53	-	-	[140,141]

Supplemental Table S1. Database of primary central nervous system (CNS) tumors outlined by the 5th edition of the World Health Organization (WHO) classification scheme and stratified by tumor category, assigned WHO grade, known epigenetic and/or genetic mutations of interest, and documented aberrant endogenous retrovirus (ERV) expression. *Abbreviations: ALK (Anaplastic lymphoma kinase); ALV (Avian leukosis virus); ARID1A (AT-Rich Interaction Domain 1A); ARID1B (AT-Rich Interaction Domain 1B); ARID2 (AT-Rich Interaction Domain 2); ATRX (Alpha-thalassemia/mental retardation, X-linked);BCOR (BCL6 Corepressor); BICRA (BRD4 Interacting Chromatin Remodeling Complex Associated Protein); CIC (Capicua transcriptional repressor); CREBBP (cAMP-response element binding protein); CTNNB1 (Catenin beta-1); EED (Embryonic Ectoderm Development); EP300 (E1A-associated protein p300); ERV (Endogenous retrovirus); ERV3 (Endogenous retrovirus group 3); ERV9 (Endogenous retrovirus group 9); ERVK24 (Endogenous retrovirus group K member 24); ERVK3-1(Endogenous retrovirus group 3 member 1); EZHIP (Enhancer of Zeste Homologs Inhibitory Protein); FOXR2 (Forkhead Box R2); GLTSCR1 (Glioma tumor suppressor candidate region gene 1); H3K27 (Histone 3 on lysine 27); H3K36 (Histone 3 on lysine 36); H3G34 (Histone 3 on arginine 34); HERV1 (Human endogenous retrovirus group 1); HERVK (Human endogenous retrovirus group K); HERVL (Human endogenous retrovirus group L); HML-6 (Human endogenous MMTV-like 6); IDH (Isocitrate dehydrogenase); Ik1 (Ikaros 1); Ik2 (Ikaros 2); JMJD1C (Jumonji domain containing 1C); KLF4 (Kruppel-like factor 4); LIN28A (Lin-28 homolog A); MALT (Mucosa associated lymphoid tissue); MAPK (Mitogen-activated protein kinase); MMVL30 (Mouse murine leukemia virus group L member 30); MN1 (Meningioma (disrupted in balanced translocation) 1); MYBL1 (MYB proto-oncogene like 1); NCoA-2 (Nuclear receptor coactivator 2); NEC (Not elsewhere classified); NK (Natural Killer); NOS (Not otherwise specified); NOTCH (Neurogenic locus notch homolog protein); NR (Not Reported); NRAS (Neuroblastoma ras viral oncogene homolog); NUTM1 (NUT midline carcinoma family member 1); OCT4 (Octamer-binding transcription 4); PBRM1 (Polybromo-1); RB1 (Retinoblastoma Transcriptional Corepressor 1); RELA (V-rel reticuloendotheliosis viral oncogene homolog A); SETD2 (SET domain-containing 2); SHH (Sonic Hedgehog); SMARCA2 (SWI/SNF Related Matrix Associated, Actin Dependent Regulator of Chromatin Subfamily A, Member 2); ; SMARCA4 (SWI/SNF Related Matrix Associated, Actin Dependent Regulator of Chromatin Subfamily A, Member 4); SMARCB1 (SWI/SNF Related Matrix Associated, Actin Dependent Regulator of Chromatin Subfamily B, Member 1); SMARCE1 (SWI/SNF Related Matrix Associated, Actin Dependent Regulator of Chromatin Subfamily E, Member 1); SUZ12 (Zeste 12 homolog); SWI/SNF (SWItch/Sucrose Non-Fermentable); TP53 (Tumor protein 53); WNT (Wingless-related integration site); YAP1 (Yes-associated protein-1); ZFTA (Zinc finger translocation associated)*

[1]reported in one recurrent case with glioblastoma-like histology
[2]reported in rare tumors with anaplastic features
[3]dependent on composition of tumor-subtypes contained within primary tumor
[4]reported in meningiomas without concomitant Merlin mutation
[5]reported only in pituitary carcinomas

Primary CNS Tumors by WHO Classification Diagnosis	WHO Grade(s)	Pathways Interacting with SWI/SNF	Human ERV(s) Expressed	Non-Human ERV(s) Expressed	References
**Gliomas, glioneuronal tumors, and neuronal tumors**					
Adult-type diffuse gliomas					
Glioblastoma, IDH-wildtype	4	EZHIP, SETD2; TP53; MYC; RB1	HERV1, HERVK, HERVL, ERV3, HML-6 (ERVK3-1)	-	[28,29,78,79,88]
**Choroid plexus tumors**					

Choroid plexus papilloma	1	TP53 (rare)	-	Roux Sarcoma Virus	[5,104–106]
**Embryonal tumors**					
Other CNS embryonal tumors					
Atypical teratoid/rhabdoid tumor	4	SHH, NRAS, MYC	HERV-K	-	[5,10,45–50]
**Cranial and paraspinal nerve tumors**					
Malignant peripheral nerve sheath tumor	NR	SUZ12, EED, H3K27; TP53	-	ALV	[61–66,115–117]
**Meningioma**					
Secretory meningioma	1-3	KLF4	HERV-K[4]	-	[59,118]
Atypical meningioma	2-3	TP53	HERV-K[4]	-	[59,119]
Anaplastic (malignant) meningioma	3	H3K27M; TP53	HERV-K[4]	-	[5,59,119]
**Mesenchymal, non-meningothelial tumors involving the CNS**					
Soft tissue tumors: Tumors of uncertain differentiation					
Ewing sarcoma	4	FET; TP53	Syncytin-1; ERV-L	-	[80,123–126]
**Melanocytic tumors**					
Circumscribed meningeal melanocytic neoplasms					
Melanocytoma and melanoma	NR	YAP1	-	MMVL30	[81,131]
**Hematolymphoid tumors involving the CNS**					
Lymphomas: CNS lymphomas					
Primary diffuse large B-cell lymphoma of the CNS	NR	MYC	Variety - Unspecified	-	[82,132]
**Germ cell tumors**					
Mature teratoma	NR	JMJD1C; RB1	ERVK24	-	[83,134,135]
Germinoma	NR	JMJD1C, LIN28A; TP53; RB1	ERVK24	-	[83,134–136]
Embryonal carcinoma	NR	JMJD1C, LIN28A; RB1	ERVK24	-	[83,134,135]
Yolk sac tumor	NR	JMJD1C, LIN28A; TP53; RB1	ERVK24	-	[83,134–136]
Choriocarcinoma	NR	JMJD1C; RB1	ERVK24	-	[83,134,135]

Supplemental Table S2. Primary CNS tumors with known genetic and/or epigenetic altered pathways with direct interaction with SWI/SNF subunits and aberrant ERV expression implicated in their tumorigenesis.
